# Risky sexual behaviours and associated factors among high school students in Lomé, Togo: A cross-sectional study

**DOI:** 10.1371/journal.pone.0335879

**Published:** 2026-08-14

**Authors:** Essi Edjodjinam Kpegba-Fiaboe

**Affiliations:** Regional Institute for Population Studies (RIPS), College of Humanities, University of Ghana, Legon, Accra, Ghana; Federal Medical Centre Birnin Kudu, NIGERIA

## Abstract

**Background:**

Adolescent sexual and reproductive health remains a major public health concern in sub-Saharan Africa. In Togo, evidence on risky sexual behaviours (RSBs) among in-school adolescents is limited. This study examined patterns of RSB and associated sociodemographic, relational, and digital factors among high school students in Lomé.

**Methods:**

A cross-sectional study was conducted from December 2023 to March 2024 among 461 high school students aged 15–23 years. Participants were selected through multistage sampling and completed a structured, self-administered questionnaire. RSB was defined as reporting at least one of the following: inconsistent condom use, overlapping sexual partnerships, substance-influenced sex, or casual sexual encounters. Descriptive statistics and logistic regression analyses were used to examine patterns and associated factors.

**Results:**

Nearly half of respondents (47.3%) reported sexual intercourse during the past 12 months. Among sexually active students, 58.7% reported sexual debut before age 18, 56.4% inconsistent condom use, 40.8% overlapping sexual relationships, and 27.5% coerced first sex. Gendered patterns were observed, with females more likely to report coerced first sex, older partners, and transactional sex. In adjusted analyses, higher odds of RSB were associated with personal mobile phone ownership (AOR = 11.35, 95% CI: 3.11–41.34), sexual debut at age 18 or older (AOR = 5.54, 95% CI: 1.55–19.82), and coerced first sex (AOR = 5.84, 95% CI: 1.15–29.49). Lower odds were observed among students with one to two agemates in the household (AOR = 0.23, 95% CI: 0.05–0.98), those identifying with other religions (AOR = 0.07, 95% CI: 0.01–0.37), and those living alone (AOR = 0.04, 95% CI: 0.00–0.39).

**Conclusions:**

RSBs among high school students in Lomé highlight gaps in sexual health literacy, consent negotiation, condom-use skills, and online safety. Strengthening comprehensive sexuality education, integrating digital literacy into school curricula, and expanding gender-responsive, youth-friendly SRH interventions are essential to promote safer, informed, and equitable adolescent sexual practices in Togo.

## Introduction

Adolescence represents a pivotal developmental stage characterized by rapid biological maturation, evolving cognitive capacities, and expanding social autonomy. It is during this period that individuals begin to consolidate their identity, assert independence, and navigate increasingly complex interpersonal and sexual relationships [1–3]. As adolescents strive for autonomy, they also experience a profound need for social belonging, rendering them highly responsive to peer norms and social expectations [4]. This dynamic tension between individuation and conformity heightens vulnerability to external pressures, such as misinformation, social coercion, and unequal gender or power relations that can manifest in risky sexual behaviour (RSB) [3,5].

Globally, adolescent sexual and reproductive health (SRH) remains a major public health concern. At regional and national levels, several policy commitments recognize the need to protect adolescents’ health and rights, including the *African Charter on the Rights and Welfare of the Child* [6] and Togo’s *Code de l’Enfant* (Law No. 2007–017), which promotes the protection of children’s rights and defines a child as any person under 18 years. Despite these commitments, adverse SRH outcomes, including early sexual debut, unintended pregnancy, sexually transmitted infections (STIs), and unsafe abortions, remain high, particularly in low- and middle-income countries (LMICs) [7,8]. The World Health Organization [9] estimates that approximately 21 million girls aged 15–19 years become pregnant annually, with nearly half of these pregnancies unintended. In sub-Saharan Africa (SSA), one in four adolescent girls gives birth before the age of 18 [10], often resulting in early school dropout, limited socioeconomic mobility, and intergenerational cycles of poverty and gender inequality [11–13].

Across West Africa, structural and sociocultural determinants such as restrictive gender norms, economic precarity, and limited access to youth-friendly SRH services, continue to drive unsafe sexual practices [11,14]. Early sexual initiation, frequently before age 15, coupled with inconsistent contraceptive use, multiple or concurrent partnerships, and transactional relationships remain common among adolescents [8,15–18]. Moreover, silence around sexuality in families and schools restricts open communication, leaving young people to rely on peers or digital media, often sources of misinformation [19–21].

In Togo, national data reflect these regional trends. The Ministry of Health reports that 17% of Togolese girls aged 15–19 have been pregnant or have given birth, with the highest prevalence in the Kara (25.3%) and Maritime (19.0%) regions [22]. According to the Togolese Ministry of Education, over 8,600 pregnancies were recorded among pre-university students nationwide (2020–2023), including 2,025 among senior high school girls [23]. Local studies confirm high rates of early sexual initiation and low contraceptive uptake among adolescents [24–26]. Limited parent–child communication around sexuality [27–29] and the stigmatization of contraceptive use further exacerbate these outcomes [24,30,31], leading to unplanned pregnancies, unsafe abortions, and serious reproductive health complications such as haemorrhage, sepsis, and, in severe cases, maternal death [32].

Beyond the risk of pregnancy and its social consequences, RSBs contribute to a significant burden of sexually transmitted infections, including HIV, among adolescents [33,34]. Yet, empirical research in Togo has predominantly focused on university students or out-of-school youth, leaving high school students, a group navigating the intersection of puberty, peer influence, and emerging digital exposure, largely understudied [35,36]. Moreover, existing literature tends to emphasize outcomes rather than determinants, limiting understanding of the behavioural and contextual factors shaping adolescent sexuality in this setting.

The present study addresses this critical gap by examining patterns of RSB and associated factors among high school students in Lomé, Togo. Specifically, it explores early sexual initiation, sexual partnership dynamics, condom use, substance-influenced sex, and transactional encounters, alongside key sociodemographic and contextual correlates. By elucidating these relationships, this study provides empirical evidence to inform youth-centred interventions and comprehensive sexuality education programs aligned with Sustainable Development Goal 3.7, which seeks to ensure universal access to sexual and reproductive health information, education, and services by 2030.

## Methods

### Study design and setting

This study employed a cross-sectional design and was conducted between December 4^th^, 2023, and March 30^th^, 2024, in five high schools in Lomé, Togo, the country’s capital and largest urban centre. The selected schools included both public and private institutions, ensuring representation across varying socioeconomic and educational contexts. Lomé was chosen as the study area because of its dense adolescent population, growing digital connectivity, and diversity in school types, which together capture diverse urban school-based adolescent experiences.

### Eligibility criteria

Eligible participants were students aged 15–24 years who were enrolled in one of the selected high schools during the study period and were able to understand French or at least one of the local languages used during data collection. Otherwise eligible students were excluded if illness prevented them from completing the questionnaire, if they declined to participate, or if they withdrew their consent or assent.

### Sample size determination

The minimum required sample size was calculated using Cochran’s formula [37] for cross-sectional studies with an unknown population size:


$$\begin{array}{c} n_{\text{0}}=\frac{Z^{\text{2}}p(\text{1}-p)}{e^{\text{2}}} \end{array}$$

where:

Z=1.96 corresponds to a 95% confidence level,

p=0.5 (assumed proportion of RSB among the study participants), and

e=0.05 (margin of error).

The initial sample size obtained was 384. To account for potential non-response or incomplete data (20%), the final sample size was adjusted to 461 participants, which was achieved during data collection.

### Data collection instruments and procedure

A multistage sampling approach was employed for this study. In the first stage, five high schools in Lomé were purposively selected based on size, type (public/private), and accessibility to ensure socioeconomic and institutional diversity. In the second stage, a proportionate stratified sampling method determined the number of participants from each school according to student enrolment. Within each school, simple random sampling was used to select individual respondents from class registers, ensuring balanced representation by sex and grade level (10^th^ –12^th^).

Data were collected using a structured, self-administered questionnaire adapted from validated adolescent SRH surveys [38–40]. The sexual initiation and first sexual experience section assessed recent sexual activity by asking participants whether they had engaged in sexual intercourse during the past 12 months. This item was used to identify respondents who were sexually active. Among those who reported being sexually active, additional questions assessed age at first sexual intercourse and the nature of the first sexual experience, categorized as *willing*, or *coerced (unwilling)*. These questions aimed to identify the timing and consent dynamics surrounding sexual debut.

The third section on sexual partnerships and relationship patterns focused on the number of partners in the past 12 months, and the presence of overlapping (concurrent) sexual relationships. Additional questions examined age disparity with the most recent sexual partner (same age, 3–5 years older, ≥ 10 years older) and engagement in transactional sex (exchange of money, gifts, or material items for sex). Finally, sexual practices and contexts of sexual activity section captured protective and risk behaviours, including condom use frequency (“always,” “sometimes,” “never”), reasons for inconsistent use, sexual activity under the influence of alcohol or drugs, and experiences of casual or one-time sexual encounters or overlapping relationship. Participants were also asked about exposure to pornography to contextualize broader behavioural pattern associated with sexual risk-taking. The questionnaire was pre-tested among 30 students from a non-participating school in Lomé to evaluate clarity, comprehension, and cultural sensitivity. Feedback from this pilot test informed revisions to wording and structure to improve reliability and response accuracy.

Four trained research assistants fluent in French and local languages (Ewé and Kabyè) administered the questionnaires during scheduled school hours. The survey was conducted in classrooms, with participants completing the anonymous forms individually under supervision to ensure privacy and minimize peer influence. Respondents handed the completed questionnaires in sealed envelopes directly to the researchers to maintain confidentiality. All filled questionnaires were checked daily for completeness and consistency before data entry.

In this study, RSB, the primary outcome variable, was measured using a composite dependent variable comprising four key behavioural indicators: (1) overlapping or concurrent sexual partnerships, (2) inconsistent condom use, (3) sexual activity under the influence of substances, and (4) casual (one-time) sexual encounters. Participants who reported engaging in at least one of these behaviours were categorized as exhibiting “any risky sexual behaviour”, resulting in a binary outcome variable coded as *0 = No* and *1 = Yes*. This dependent variable was tested for association with socio-demographic factors, including age group, sex, grade level, religion, living arrangement, number of agemates in the household, engagement in paid work, and mobile device ownership. Additionally, behavioural and contextual factors, such as age at sexual debut, nature of first sexual experience, number of sexual partners in the past 12 months, partner age difference, engagement in transactional sex, and exposure to pornography, were also analyzed to account for variations in sexual behaviour patterns and relational context.

### Statistical analysis

Data were entered, cleaned, and coded in Microsoft Excel and exported to Stata version 17 for analysis. Descriptive statistics, including frequencies, percentages, means, and standard deviations, were used to summarize participants’ sociodemographic characteristics and sexual behaviour patterns. Binary logistic regression was used to examine associations between RSB and the independent variables. Bivariate logistic regression analyses were first conducted to assess the association between each independent variable and RSB. Variables considered relevant were subsequently included in the multivariable logistic regression model to identify independent predictors of RSB. Crude odds ratios (CORs) and adjusted odds ratios (AORs), with 95% confidence intervals (CIs), were estimated to determine the strength and direction of associations. Statistical significance was set at *p* < 0.05.

### Ethical considerations

The study was conducted in full adherence to national and international ethical standards for research involving human participants. Ethical approval was obtained from the National Ethics Committee for Health Research in Togo (Protocol No. 047/2023/CBRS) and the Ethics Committee for the Humanities, University of Ghana (Approval No. ECH 285/22–23). Additional authorization was granted by the Ministry of Primary, Secondary, and Technical Education in Togo (Ref. 2454-23/DRE-GL), the Regional Directorate of Education, and the respective school administrations. Written informed consent was obtained from all participants aged 18 years and above, while assent and parental consent were obtained for those under 18. Only students selected through the sampling procedure and who provided consent or assent completed the questionnaire; non-selected or non-consenting students did not participate. To protect participants’ privacy and confidentiality, each respondent was assigned a unique identification code; no names or identifiable details were recorded. Data were securely stored in password-protected files accessible only to the research team. Although the study posed no foreseeable risks, a trained mental health professional was available during data collection to provide immediate support to participants who might have experienced distress while responding to questions on sensitive sexual and reproductive health topics.

### Inclusivity in global research

Additional information regarding the ethical, cultural, and scientific considerations specific to inclusivity in global research is included in the Supporting Information (S1 Checklist).

## Results

### Socio-demographic characteristics

A total of 461 high school students participated in the study (Table 1). Slightly more than half of the respondents were male (53.8%), while females accounted for 46.2%. The majority (89.1%) were aged between 15 and 18 years, with a mean age of 16.9 years (SD = 2.0). In terms of grade level, 37.5% were in 10^th^ grade, 33.6% in 11^th^ grade, and 28.9% in 12^th^ grade. Regarding household composition, about 62% reported living with more than three agemates, while 22% lived with one or two, and 16% had no agemates at home. Christianity was the predominant religion among respondents (47.7%), followed by Islam (41.2%), while 11.1% reported as animists or having no religious affiliation. Nearly half (47.5%) of participants lived with both parents, while 37.7% lived with a single parent (biological father or mother), 11.7% with other relatives or non-relatives, and 3.1% reported living alone. In terms of economic engagement, the majority (69.4%) had never engaged in paid casual work, 19.1% did so occasionally, and 11.5% reported working often. Concerning mobile device ownership, 41.4% of respondents primarily accessed a peer’s device (shared phone use), while 58.6% owned a personal mobile device.

**Table 1:** Socio-demographic characteristics of high school students in Lomé, Togo, 2024 (*N* = 461).

Variable	Category	Frequency (*N* = 461)	Percentage
Sex	Male	248	53.8
	Female	213	46.2
Age group	15–18	411	89.1
	19–23	50	10.9
Mean age (±SD)	16.9 ± 2.0	–	–
Grade	10^th^ Grade	173	37.5
	11^th^ Grade	155	33.6
	12^th^ Grade	133	28.9
Agemates in household^*a*^	None	73	15.8
	1–2	103	22.4
	3–4	184	39.9
	5+	101	21.9
Religion	Christianity	220	47.7
	Islam	190	41.2
	Other religions^*b*^	41	11.1
Living arrangement	Both parents	219	47.5
	Single parent	174	37.7
	Other relative/non-relative	54	11.7
	Living alone	14	3.1
Paid casual work	Never	320	69.4
	Occasionally	88	19.1
	Often	53	11.5
Mobile device ownership	Peer’s device^*c*^	191	41.4
	Personal device	270	58.6

^a^ Agemates in household refer to other individuals of similar age living in the same household.

^b^ “Other religion” includes respondents who identified with traditional beliefs (e.g., Animism) or reported having no religious affiliation.

^c^ Peer’s device refers to a mobile phone belonging to a friend or classmate that the respondent uses.

### Sexual behaviour and related experiences

Out of the total sample of 461 respondents, nearly half (47.3%) reported sexual intercourse during the past 12 months and were therefore classified as sexually active (Table 2). Sexual activity differed significantly by sex, with a higher proportion of females reporting past-year sexual intercourse compared with males (54.5% vs. 41.1%; *p* = 0.004).

**Table 2:** Sexual and other related experiences among sexually active high school students in Lomé, Togo, 2024 (*n* = 218).

Variable	Category	Male (n = 102)	Female (n = 116)	Total(n = 218)^*a*^	*P*-value^*b*^
Sexually active (*N* = 461)^*c*^	No	146 (58.9)	97 (45.5)	243 (52.7)	0.004
	Yes	102 (41.1)	116 (54.5)	218 (47.3)	
Age at sexual debut^*d*^	Before age 18	67 (65.7)	61 (52.6)	128 (58.7)	0.050
	18 + years	35 (34.3)	55 (47.4)	90 (41.3)	
First sexual experience	Willing	95 (93.1)	63 (54.3)	158 (72.5)	<0.001
	Coerced	7 (6.9)	53 (45.7)	60 (27.5)	
12-month partners^*e*^	Single	56 (54.9)	49 (42.2)	105 (48.2)	0.062
	Multiple	46 (45.1)	67 (57.8)	113 (51.8)	
Partner age difference^*f*^	~Same age	88 (86.3)	58 (50.0)	146 (67.0)	<0.001
	10 + years older	14 (13.7)	58 (50.0)	72 (33.0)	
Transactional sex^*g*^	Never had	92 (90.2)	62 (53.5)	154 (70.6)	<0.001
	Ever had	10 (9.8)	54 (46.5)	64 (29.4)	
Watch porn media	Never	21 (20.6)	28 (24.1)	49 (22.5)	0.499
	Occasionally	53 (52.0)	51 (44.0)	104 (47.7)	
	Often	28 (27.4)	37 (31.9)	65 (29.8)	
Overlapping sexual relationships	Never had	62 (60.8)	67 (57.8)	129 (59.1)	0.883
	Ever had	40 (39.2)	49 (42.2)	89 (40.8)	
Condom use consistency	Consistent	42 (41.2)	53 (45.7)	95 (43.6)	0.503
	Inconsistent	60 (58.8)	63 (54.3)	123 (56.4)	
Substance-influenced sexual activity	Never had	92 (90.2)	102 (87.9)	194 (89.0)	0.594
	Ever had	10 (9.8)	14 (12.1)	24 (11.0)	
Casual (one-time) sexual encounters	Never had	74 (72.6)	99 (85.3)	173 (79.4)	0.020
	Ever had	28 (27.4)	17 (14.7)	45 (20.6)	

^a^ Sexually active in the past 12 months (*N* = 461).

^b^ P-values compare male and female respondents.

^c^ Sexually active participants were respondents who reported sexual intercourse during the past 12 months.

^d^ Age at sexual debut refers to the respondent’s age at first sexual intercourse.

^e^ “12-month partners” indicates the number of sexual partners reported in the past 12 months.

^f^ Partner age difference represents the age gap between the respondent and their most recent sexual partner.

^g^ Transactional sex refers to engaging in sexual relationships in exchange for money, gifts, or other material benefits.

Among those who were sexually active (*n* = 218), more than half (58.7%) reported initiating sexual activity before the age of 18, whereas 41.3% experienced sexual debut at 18 years or older. Early sexual debut was more common among males than females (65.7% vs. 52.6%; *p* = 0.050). Most respondents described their first sexual experience as willing (72.5%), whereas 27.5% reported that their first sexual experience was coerced. This differed markedly by sex: 45.7% of females reported coerced first sex compared with 6.9% of males (*p* < 0.001).

Regarding recent sexual partnerships, 48.2% of sexually active respondents reported having a single sexual partner in the past 12 months, while 51.8% reported multiple partners. Two-thirds of respondents (67.0%) reported that their most recent sexual partner was approximately the same age, whereas 33.0% reported having a partner who was at least 10 years older. Females were substantially more likely than males to report an older partner (50.0% vs. 13.7%; *p* < 0.001). Transactional sex was reported by 29.4% of sexually active respondents and was significantly more common among females than males (46.5% vs. 9.8%; *p* < 0.001). Regarding exposure to sexually explicit content, almost half of the respondents (47.7%) indicated watching pornographic media occasionally, 29.8% reported frequent viewing, and 22.5% stated they had never watched such content. Pornography exposure did not differ significantly by sex (*p* = 0.499).

With respect to the specific indicators of risky sexual behaviour, 40.8% of sexually active respondents reported having ever been involved in overlapping or concurrent sexual relationships, with no significant difference between males and females (39.2% vs. 42.2%; *p* = 0.883). Consistent condom use was reported by 43.6% of respondents, while 56.4% reported inconsistent condom use. Condom use consistency did not differ significantly by sex (*p* = 0.503). Substance-influenced sexual activity was reported by 11.0% of respondents, with similar proportions among males and females (9.8% vs. 12.1%; *p* = 0.594). Casual or one-time sexual encounters were reported by 20.6% of sexually active respondents and were significantly more common among males than females (27.4% vs. 14.7%; *p* = 0.020).

### Factors associated with risky sexual behaviour

In the bivariate model, students in higher grades were more likely to report RSB compared to those in 10^th^ grade (COR = 3.20; 95% CI: 1.42–7.24; *p* = 0.005; Table 3). Students with one to two agemates in the household had lower odds of RSB than those with no agemates (COR = 0.28; 95% CI: 0.10–0.81; *p* = 0.019). Other religions were also associated with lower odds of RSB compared with Christianity (COR = 0.29; 95% CI: 0.09–0.91; *p* = 0.035), while living with a single parent was associated with higher odds compared with living with both parents (COR = 2.27; 95% CI: 1.04–4.95; *p* = 0.039). Personal mobile phone ownership was strongly associated with RSB in the crude model (COR = 9.83; 95% CI: 4.54–21.25; *p* < 0.001).

**Table 3:** Factors associated with risky sexual behaviour (RSB) among sexually active high school students in Lomé, Togo, 2024 (*n* = 218).

Variable	RSB n (%)		COR (95% CI)	AOR (95% CI)
	No(*n* = 49)	Yes(*n* = 169)		
**Sex** ^[Male]^	22 (21.6)	80 (78.4)	1	1
Female	27 (23.3)	89 (76.7)	0.90 (0.47–1.71)	0.42 (0.12–1.50)
**Age** ^[15–18]^	46 (24.7)	140 (75.3)	1	1
19–23	3 (9.4)	29 (90.6)	3.17 (0.92–10.91)	2.16 (0.29–16.00)
**Grade** ^[10th]^	23 (34.8)	43 (65.2)	1	1
11^th^	15 (20.0)	60 (80.0)	2.13 (1.00–4.57)	1.88 (0.56–6.34)
12^th^	11 (14.3)	66 (85.7)	3.20** (1.42–7.24)	1.32 (0.36–4.80)
**Agemates in household** ^[None]^	5 (9.8)	46 (90.2)	1	1
1–2	24 (27.3)	64 (72.7)	0.28* (0.10–0.81)	0.23 (0.05–0.98)
3–4	14 (24.6)	43 (75.4)	0.33 (0.11–1.01)	0.41 (0.09–1.86)
5+	6 (27.3)	16 (72.7)	0.28 (0.08–1.08)	0.57 (0.09–3.60)
**Religion** ^[Christianity]^	38 (20.0)	152 (80.0)	1	1
Islam	5 (33.3)	10 (66.7)	0.49 (0.16–1.54)	0.44 (0.08–2.25)
Other religions	6 (46.2)	7 (53.8)	0.29* (0.09–0.91)	0.07** (0.01–0.37)
**Living arrangement** ^[Both parents]^	25 (25.5)	73 (74.5)	1	1
Single parent	11 (13.1)	73 (86.9)	2.27* (1.04–4.95)	2.11 (0.65–6.77)
Other relative/nonrelative	8 (34.8)	15 (65.2)	0.64 (0.24–1.69)	0.31 (0.07–1.36)
Alone	5 (38.5)	8 (61.5)	0.54 (0.16–1.83)	0.04** (0.00–0.39)
**Paid casual work** ^[Never]^	35 (25.0)	105 (75.0)	1	1
Occasionally	9 (18.0)	41 (82.0)	1.51 (0.67–3.43)	1.16 (0.33–4.06)
Often (at least once a month)	5 (17.9)	23 (82.1)	1.53 (0.54–4.33)	0.86 (0.21–3.46)
**Mobile device ownership** ^[No personal phone]^	39 (44.8)	48 (55.2)	1	1
Personal phone	10 (7.6)	121 (92.4)	9.83*** (4.54–21.25)	11.35*** (3.11–41.34)
**Age at sexual debut** ^[Before age 18]^	40 (31.3)	88 (68.7)	1	1
18 + years	9 (10.0)	81 (90.0)	4.09*** (1.86–8.95)	5.54** (1.55–19.82)
**First sexual experience** ^[Willing]^	45 (28.5)	113 (71.5)	1	1
Coerced	4 (6.7)	56 (93.3)	5.57** (1.90–16.27)	5.84* (1.15–29.49)
**12-month partners** ^[Single]^	33 (31.4)	72 (68.6)	1	1
Multiple	10 (11.8)	75 (88.2)	2.77** (1.42–5.43)	2.11 (0.74–6.01)
**Partner age difference** ^[~Same age]^	37 (25.3)	109 (74.7)	1	1
10 + years older	12 (16.7)	60 (83.3)	1.69 (0.82–3.49)	0.99 (0.29–3.37)
**Transactional sex** ^[No]^	36 (23.4)	118 (76.6)	1	1
Yes	13 (20.3)	51 (79.7)	1.19 (0.58–2.44)	0.82 (0.24–2.74)
**Pornography** ^[Never]^	25 (51.0)	24 (49.0)	1	1
Occasionally	19 (18.3)	85 (81.7)	4.66*** (2.20–9.85)	2.50 (0.78–7.95)
Often	5 (7.7)	60 (92.3)	12.49*** (4.28–36.46)	4.79 (0.82–27.79)
***Pseudo R2***			***0.445***	
***Model p***			***<0.0001***	
***Hosmer–Lemeshow test***			***χ² = 3.00, p = 0.934***	

^a^ [Superscript] indicates reference categories for comparison.

*p < 0.05; ** *p* < 0.01; *** *p* < 0.001.

Sexual and behavioural factors also showed important crude associations with RSB. Students who initiated sex at age 18 or older had higher odds of RSB than those who initiated before age 18 (COR = 4.09; 95% CI: 1.86–8.95; *p* < 0.001). Those whose first sexual experience was coerced had higher odds of RSB than those whose first sexual experience was willing (COR = 5.57; 95% CI: 1.90–16.27; *p* = 0.002). Multiple sexual partnerships in the past 12 months were also associated with higher odds of RSB compared with having a single partner (COR = 2.77; 95% CI: 1.42–5.43; *p* = 0.003). Pornography exposure showed a dose-related crude association: occasional viewers had higher odds of RSB than those who never watched pornography (COR = 4.66; 95% CI: 2.20–9.85; *p* < 0.001), while frequent viewers had substantially higher odds (COR = 12.49; 95% CI: 4.28–36.46; *p* < 0.001).

After adjustment, fewer variables remained significantly associated with RSB. Students with one to two agemates in the household had lower odds of RSB than those with no agemates (AOR = 0.23; 95% CI: 0.05–0.98; *p* = 0.047). Respondents identifying with other religions had lower odds of RSB than Christians (AOR = 0.07; 95% CI: 0.01–0.37; *p* = 0.002), and those living alone had lower odds than those living with both parents (AOR = 0.04; 95% CI: 0.00–0.39; *p* = 0.005). Personal mobile phone ownership remained a strong independent predictor of RSB, with students who owned a phone having more than eleven times the odds of RSB compared with those who did not own one (AOR = 11.35; 95% CI: 3.11–41.34; *p* < 0.001).

Regarding sexual history, students who initiated sex at age 18 or older had higher odds of RSB than those who initiated before age 18 (AOR = 5.54; 95% CI 1.55–19.82; *p* = 0.008). Similarly, students whose first sexual experience was coerced had higher odds of RSB than those whose first sexual experience was willing (AOR = 5.85; 95% CI 1.15–29.49; *p* = 0.032). In contrast, sex, age group, grade level, paid casual work, number of sexual partners, partner age difference, transactional sex, and pornography exposure were not significantly associated with RSB after adjustment (*p* > 0.05; Table 3).

COR = Crude Odds Ratio; AOR = Adjusted Odds Ratio; CI = Confidence Interval.

## Discussion

This study examined patterns of risky sexual behaviour and associated factors among high school students in Lomé, Togo. Nearly half of respondents reported sexual intercourse during the past 12 months, and several sexual risk-related experiences were common among sexually active students, including early sexual debut, inconsistent condom use, multiple partnerships, overlapping sexual relationships, and coerced first sex. In the adjusted analysis, RSB was independently associated with later sexual debut, coerced first sexual experience, and personal mobile phone ownership. Lower odds of RSB were observed among students with one to two agemates in the household, those identifying with religions other than Christianity, and those living alone. These findings suggest that adolescent sexual risk in this setting is shaped by intersecting relational, household, gendered, and digital influences.

Early sexual initiation was reported by more than half of sexually active participants, consistent with findings from other urban populations across SSA, where sexual debut before the age of 18 remains widespread [8,41–43]. In this study, early sexual debut was more common among males than females, while females more often reported coerced first sex and older sexual partners, suggesting that the timing of sexual initiation may be shaped by different gendered pathways of autonomy, pressure, and relational power. Early sexual debut remains concerning because it has been linked to unprotected intercourse, multiple partnerships, sexually transmitted infections, unintended pregnancy, and other adverse sexual and reproductive health outcomes [15,43,44]. Intriguingly, however, adolescents in the present study who initiated sex at age 18 or older were significantly more likely to engage in RSB than those who initiated before age 18. This finding contrasts with much of the existing literature, including multi-country evidence showing that early sexual debut is associated with adverse sexual and relational outcomes such as greater number of sexual partners and reduced sexual protective measures [18,45–47]. One possible explanation is that later initiation may occur among older students with greater autonomy, wider peer and romantic networks, and increased mobility [48], but without adequate sexual health literacy, condom negotiation skills, or access to youth-friendly SRH services. For female students, later sexual debut may also occur within unequal relationships involving older partners or coercive dynamics [49]. Thus, delayed sexual debut may not necessarily be protective when it occurs in contexts of limited preparedness, constrained agency, or gendered power imbalance.

Notably, more than half of the sexually active participants in this study reported having multiple sexual partners in the 12 months preceding data collection, and two in five acknowledged overlapping relationships. Although overlapping relationships did not differ significantly by sex, these partnership patterns can be interpreted within broader gendered relationship dynamics, particularly given the higher reporting of older partners, transactional sex, and coerced first sex among female students. Multiple and concurrent partnerships are concerning because they broaden young people’s sexual exposure network and increase the likelihood of unintended pregnancies and STIs, including HIV [50–53]. Studies reporting similar findings from other urban African settings have shown that such relationships often occur within contexts marked by inconsistent condom use, emotional instability, and unequal power dynamics, which may further constrain adolescents’ ability to negotiate safer sex [11,54,55]. These findings reinforce the need for interventions that address not only behavioural risk, but also the relational, psychosocial, and gendered conditions that sustain high-risk sexual networks among young people.

In addition to behavioural experimentation, relational power dynamics emerged as an important factor associated with adolescent sexual risk. More than one-quarter of sexually active participants reported coerced first sex, with a markedly higher proportion among females than males. Those who reported coerced first sex had nearly six times the odds of RSB compared with those whose first sexual experience was willing. This finding suggests that early coercive experiences may undermine sexual agency, weaken adolescents’ ability to negotiate consent and condom use, and increase vulnerability to subsequent risk exposure [56,57]. The lingering psychosocial impacts of coercion (such as internalized disempowerment, lowered self-worth, and normalization of non-consensual experiences) can entrench cycles of vulnerability, as supported by prior studies [58,59]. These patterns reflect broader gendered power relations in which adolescent girls may be socialized into more submissive sexual roles, while boys are often afforded greater decision-making power in intimate relationships. Such imbalances can expose girls to sexual pressure, harassment, and embarrassment around condom acquisition, thereby reducing their bargaining power in sexual and reproductive health decision-making [60–62].

These findings highlight the need for comprehensive, gender-transformative sexuality education that goes beyond biological instruction to address consent, power dynamics, condom negotiation, emotional wellbeing, and respectful relationships [63]. Although Togo has legal protections such as the *Code de l’Enfant* (Law No. 2007–017) and commitments under the *African Charter on the Rights and Welfare of the Child*, the continued reporting of coerced first sexual experiences points to gaps in implementation and persistent sociocultural barriers to gender equity [6,64,65]. Strengthening confidential school-based reporting systems, youth advocacy networks, and community sensitization initiatives is therefore essential for safeguarding adolescents’ sexual and reproductive rights.

Beyond interpersonal and household factors, the findings also point to the growing role of digital exposure in adolescent sexual risk. Personal mobile phone ownership was strongly associated with RSB, while frequent pornography exposure was common among sexually active students. Although pornography exposure did not remain independently associated with RSB after adjustment, its crude association and high prevalence suggest that it remains relevant within the broader digital environment shaping adolescent sexuality. These findings are consistent with emerging evidence from LMICs showing that digital connectivity can increase access to sexual health information while also exposing adolescents to sexually explicit content, peer pressure, and online solicitation [20,66,67]. They also align with studies linking pornography exposure to distorted sexual norms, objectification, and risky sexual practices [68–70]. These patterns reflect a “digital double bind”: smartphones may empower adolescents by expanding access to information and social connection, yet they may also provide unfiltered access to sexualized content and unsupervised online interactions. In urban settings such as Lomé, where adolescent digital connectivity is expanding, sexual and reproductive health promotion must therefore move beyond content restriction alone. Interventions should strengthen digital resilience, including adolescents’ ability to critically assess online sexual content, recognize harmful or coercive digital interactions, resist peer pressure, and seek reliable SRH information [71,72].

At the institutional level, integrating digital literacy and online safety into secondary school curricula could complement comprehensive sexuality education. Partnerships between education, health, digital technology, and youth sectors could support moderated online SRH forums, verified educational social media content, parental digital guidance modules, and anonymous reporting tools for sexual violence or online harassment. Existing youth-friendly platforms such as *InfoAdoJeunes* could also be expanded to include interactive digital literacy modules and peer mentorship spaces. Community-based organizations, media regulators, mobile network operators, and youth advocates could further support ethical digital citizenship campaigns that promote respectful online behaviour and challenge coercive sexual narratives. In light of the foregoing, these approaches position digital technology not only as a potential source of risk, but also as a tool for strengthening adolescents’ sexual health literacy, agency, and resilience. Building safer digital environments will require multisectoral collaboration to ensure that Togo’s digital transition supports adolescent wellbeing and reproductive rights.

## Conclusion

This study examined risky sexual behaviours among high school students in Lomé, Togo. Among students who reported sexual intercourse during the past 12 months, RSB was independently associated with personal mobile phone ownership, later sexual debut, and coerced first sexual experience. Female students more often reported coerced first sex, older sexual partners, and transactional sex, highlighting important gendered dimensions of adolescent sexual vulnerability. These findings suggest that adolescent sexual risk in this setting is shaped by relational, household, gendered, and digital factors. They support the need for comprehensive, gender-responsive, and digitally informed sexual and reproductive health interventions for adolescents.

### Strengths and limitations of the study

This study addresses an important evidence gap by examining risky sexual behaviour among high school students in Lomé, a group that remains underrepresented in adolescent sexual and reproductive health research in Togo. It assessed multiple dimensions of sexual risk, including inconsistent condom use, overlapping partnerships, substance-influenced sex, casual sex, coerced first sex, transactional sex, partner age difference, and digital exposure. The use of a structured, pre-tested questionnaire, anonymous data collection procedures, and multivariable analysis strengthened the quality and analytical depth of the findings. Sex-disaggregated descriptive analyses also provided insight into important gendered patterns.

However, some limitations should be noted. The cross-sectional design precludes causal inference, and the use of self-reported data on sensitive behaviours may have introduced recall or social desirability bias. Although questionnaires were completed individually and anonymously, the classroom setting may not have ensured complete privacy, possibly leading to underreporting of sensitive experiences. Sexual activity was defined as intercourse during the past 12 months; therefore, adolescents with sexual experiences outside this period may not have been captured. The study also did not assess some relevant outcomes, including unintended pregnancy, pregnancy termination, sexually transmitted infections, type of sexual intercourse, and broader contraceptive use. In addition, the composite RSB measure may mask differences in the severity or context of individual behaviours. Finally, although sex-disaggregated descriptive results were presented, fully sex-stratified multivariable models were limited by sparse cells and wide confidence intervals; thus, some adjusted estimates should be interpreted cautiously.

## Supporting information

S1 Checklist Inclusivity in global research questionnaire. (DOCX)
