# Supplementary material for: Risky sexual behaviours and associated factors among high school students in Lomé, Togo: A cross-sectional study
Source: PLoS One. 2026 Aug 14;21(8):e0335879. doi: 10.1371/journal.pone.0335879 (PMC13475897; doi:10.1371/journal.pone.0335879)
Supplement: Supplementary file 2 [file pone.0335879.s002.docx]

**Patterns and determinants of risky sexual behaviours among high school students in Lomé, Togo: a cross-sectional study**

Reviewer: Charles Muruka

Date: 30 March 2026

**Overall impression**

Adolescent sexual and reproductive health remains a significant concern in public health, especially due to teenage pregnancies, unsafe abortions, STIs and HIV&AIDS. This study is important and timely as it explores determinants of risky sexual behaviours, including those that do not frequently feature in published literature i.e. digital media exposure.

**Specific comments**

1) Line 47: Policy commitments on adolescent sexual and reproductive health (ASRH) are acknowledged as existing but these are not highlighted. Please highlight all existing policy commitments, especially in Togo.
2) Lines 147-149: The survey questionnaire was administered to participants in a classroom under supervision to ensure privacy and minimize peer influence. Were all students in a classroom respondents? These needs to be clarified in the manuscript. Second, a classroom does not confer adequate privacy for respondents due to tendency of students to “peep through” or attempts to “gauge” who is ahead in completing the questionnaire. This needs to be acknowledged as a limitation under study limitations.
3) This study did not look into the self-reported outcomes of risky sexual behaviours, for example (1) incidents of unwanted pregnancies (whether these were carried to term or terminated); (2) incidents of STIs (whether they have a current or previous STI); (3) type of sex (anal, oral, vaginal); and, (4) contraceptive use. I suggest that these be highlighted and included under study limitations.
4) Did both sexes (male and female) exhibit similar or different sexual behaviours? For example, which sex is more susceptible to coerced sexual intercourse? Or engaging in transactional sex? Or not using condoms? Or substance-influenced sex? Or multiple concurrent sexual partnerships? or engagement in casual sex? Or engaging in sexual intercourse with partners 10+ years older? Etc. The analysis would be more informative if it was disaggregated by sex.
5) Line 273 (Table 3): The confidence intervals stated for agemates in the household and religion do not match the stated AORs: (1) *Agemates in the household*: the AOR of 0.23 is below the lower CI limit of 0.55; (2) *Religion*: the AOR of 0.44 is below the lower CI limit of 0.88. Please recheck the analysis.
6) Lines 278-382 (Discussion section): How do findings of this study compare and contrast with other similar studies, especially those conducted with the same region or, by extension, LMICs? Are the identified determinants of RSB similar or different and what are the most probable explanations?
7) Study limitations section is missing. Please include.

**Final remarks**

Minor revisions. Accept after minor revisions are completed.
