# Supplementary material for: Risky sexual behaviours and associated factors among high school students in Lomé, Togo: A cross-sectional study
Source: PLoS One. 2026 Aug 14;21(8):e0335879. doi: 10.1371/journal.pone.0335879 (PMC13475897; doi:10.1371/journal.pone.0335879)
Supplement: Supplementary file 3 [file pone.0335879.s004.docx]

Risky sexual behaviours and associated factors among high school students in Lomé, Togo: a cross-sectional study

Essi Edjodjinam Kpegba-Fiaboe^1,*^

^1^Regional Institute for Population Studies (RIPS), College of Humanities, University of Ghana, Legon, Accra, Ghana.

*****[eefiaboe@yahoo.com](mailto:eefiaboe@yahoo.com)

**ORCID:** 0000-0002-2511-5002

**Response to Reviewer**

I sincerely thank the reviewers for the constructive and encouraging comments on my manuscript. I have carefully revised the manuscript in response to each comment. The main revisions include strengthening the policy context, clarifying classroom-based data collection, adding sex-disaggregated descriptive analyses, correcting Table 3 estimates, expanding the Discussion to compare our findings with studies from sub-Saharan Africa and LMICs, and adding a Strengths and Limitations section.

**Comment 1**

**Line 47: Policy commitments on adolescent sexual and reproductive health are acknowledged as existing but these are not highlighted. Please highlight all existing policy commitments, especially in Togo.**

Line 48-51: I revised the Introduction to more explicitly mention relevant regional and national policy commitments. Specifically, I now refer to the African Charter on the Rights and Welfare of the Child and Togo’s *Code de l’Enfant* (Law No. 2007–017), which promotes the protection of children’s rights and defines a child as any person under 18 years. I also retained references to national adolescent pregnancy-related policy efforts and data from Togo.

**Comment 2**

**Lines 147–149: The survey questionnaire was administered to participants in a classroom under supervision to ensure privacy and minimize peer influence. Were all students in a classroom respondents? This needs to be clarified in the manuscript. Second, a classroom does not confer adequate privacy for respondents due to tendency of students to “peep through” or attempts to “gauge” who is ahead in completing the questionnaire. This needs to be acknowledged as a limitation under study limitations.**

Line 181-183: I revised the Methods section to clarify that only students selected through the sampling procedure and who provided consent or assent completed the questionnaire.

Line 397-417: I also acknowledged in the limitations that classroom-based data collection may not have ensured complete privacy, despite anonymous completion and sealed-envelope submission.

**Comment 3**

**This study did not look into the self-reported outcomes of risky sexual behaviours, for example unwanted pregnancies, STIs, type of sex, and contraceptive use. I suggest that these be highlighted and included under study limitations.**

Line 397-417: I added this point to the Strengths and Limitations section. The revised limitation now explicitly states that the study did not assess unintended pregnancy, pregnancy termination, current or previous STIs, type of sexual intercourse, or broader contraceptive use beyond condom-use consistency.

**Comment 4**

**Did both sexes exhibit similar or different sexual behaviours? The analysis would be more informative if it was disaggregated by sex.**

I revised Table 2 to present sex-disaggregated descriptive results for all key sexual behaviour variables. The revised Results section now highlights gendered patterns, including differences in past-year sexual activity, coerced first sex, partner age difference, transactional sex, and casual sexual encounters. I also expanded the Discussion to interpret these findings in relation to gendered power dynamics and sexual negotiation.

Fully sex-stratified multivariable models were explored but were limited by sparse cells and wide confidence intervals. I therefore retained the pooled multivariable model as the main analysis and noted this limitation.

**Comment 5**

**Line 273/Table 3: The confidence intervals stated for agemates in the household and religion do not match the stated AORs. Please recheck the analysis.**

I rechecked the regression outputs and corrected Table 3. The adjusted odds ratios and confidence intervals now align correctly. For example, the AOR for having one to two agemates in the household is now reported as 0.23 with a 95% CI of 0.05–0.98. For religion, the revised table reports Islam as AOR = 0.44, 95% CI: 0.08–2.25, and other religions as AOR = 0.07, 95% CI: 0.01–0.37.

**Comment 6**

**Lines 278–382/Discussion section: How do findings of this study compare and contrast with other similar studies, especially those conducted within the same region or LMICs? Are the identified determinants of RSB similar or different and what are the most probable explanations?**

Line 292-387: I substantially revised the Discussion to compare and contrast the findings with evidence from sub-Saharan Africa and LMICs. I discuss how early sexual initiation, multiple partnerships, coerced first sex, and digital exposure align with or differ from previous studies. I also provide possible explanations for findings that differed from prior literature, particularly the association between later sexual debut and RSB. In addition, I revised the language from “determinants” to “associated factors” to avoid causal overstatement given the cross-sectional design.

**Comment 7**

**Study limitations section is missing. Please include.**

Line 397-417: I have added a dedicated “Strengths and limitations of the study” section. This section summarizes the study’s strengths and acknowledges limitations related to cross-sectional design, self-reporting, classroom-based data collection, the 12-month definition of sexual activity, unmeasured SRH outcomes, the composite RSB measure, and limited power for fully sex-stratified multivariable analyses.
