# Supplementary material for: Risky sexual behaviours and associated factors among high school students in Lomé, Togo: A cross-sectional study
Source: PLoS One. 2026 Aug 14;21(8):e0335879. doi: 10.1371/journal.pone.0335879 (PMC13475897; doi:10.1371/journal.pone.0335879)
Supplement: Supplementary file 4 [file pone.0335879.s005.docx]

**Response to Reviewers**

**Risky sexual behaviours and associated factors among high school students in Lomé, Togo: a cross-sectional study**

Essi Edjodjinam Kpegba-Fiaboe^1,*^

^1^Regional Institute for Population Studies (RIPS), College of Humanities, University of Ghana, Legon, Accra, Ghana.

*****[eefiaboe@yahoo.com](mailto:eefiaboe@yahoo.com)

**ORCID:** 0000-0002-2511-5002

I sincerely thank the reviewers for their careful evaluation of the manuscript and their constructive feedback. The responses below describe the revisions made to address each comment.

**Reviewer #2**

**Comment:**
The second full document review confirms that all comments were satisfactorily addressed. Particularly, it is noteworthy that the reviewer recommendation on gender-disaggregated descriptive analysis unearthed important differences.

**Response:**
I sincerely thank the reviewer for confirming that the previous comments were satisfactorily addressed. I also appreciate the recommendation to present sex-disaggregated descriptive analyses, which helped identify important differences between male and female students and strengthened the interpretation of the findings.

**Reviewer #3**

**General comment:**
This is a very good research topic and is of great importance in this era of increasing digital influence, especially among young people and adolescents. The manuscript is also very well written.

**Response:**
I thank the reviewer for the positive assessment of the study topic and the quality of the manuscript. I also appreciate the helpful suggestions provided to improve the Methods section.

**Comment I – Study design:**
All cross-sectional studies are quantitative, so there is no need to describe the study as a “cross-sectional quantitative study.”

**Response:**
I agree with the reviewer. The wording has been revised from “cross-sectional quantitative design” to “cross-sectional design” in the Methods section under the subsection “Study design and setting.”

**Comment II – Eligibility criteria:**
The exclusion criteria are redundant because students outside the specified age range or those not enrolled in the selected schools were already outside the study population. Exclusion criteria should apply only to individuals who otherwise meet the inclusion criteria but are unable to participate because of factors such as illness or a language barrier.

**Response:**
I agree with the reviewer and have revised the “Eligibility criteria” subsection accordingly. The redundant exclusion criteria relating to age and enrolment in the selected schools have been removed. The revised text now clarifies that otherwise eligible students were excluded if illness prevented them from completing the questionnaire, if they declined to participate, or if they withdrew their consent or assent.
