# Supplementary material for: Risky sexual behaviours and associated factors among high school students in Lomé, Togo: A cross-sectional study
Source: PLoS One. 2026 Aug 14;21(8):e0335879. doi: 10.1371/journal.pone.0335879 (PMC13475897; doi:10.1371/journal.pone.0335879)
Supplement: Peer review history

# Peer review: Decision Letter 0

**DOI:** 10.1371/journal.pone.0335879.r001

**Authors:** Paul Obeng

3 Apr 2026

Dear Dr. Kpegba-Fiaboe,

Thank you for submitting your manuscript to PLOS ONE. After careful consideration, we feel that it has merit but does not fully meet PLOS ONE’s publication criteria as it currently stands. Therefore, we invite you to submit a revised version of the manuscript that addresses the points raised during the review process.

Please submit your revised manuscript by May 17 2026 11:59PM. If you will need more time than this to complete your revisions, please reply to this message or contact the journal office at plosone@plos.org. When you're ready to submit your revision, log on to https://www.editorialmanager.com/pone/ and select the 'Submissions Needing Revision' folder to locate your manuscript file.

- A letter that responds to each point raised by the academic editor and reviewer(s). You should upload this letter as a separate file labeled 'Response to Reviewers'.
- A marked-up copy of your manuscript that highlights changes made to the original version. You should upload this as a separate file labeled 'Revised Manuscript with Track Changes'.
- An unmarked version of your revised paper without tracked changes. You should upload this as a separate file labeled 'Manuscript'.

If you would like to make changes to your financial disclosure, please include your updated statement in your cover letter. Guidelines for resubmitting your figure files are available below the reviewer comments at the end of this letter.

If applicable, we recommend that you deposit your laboratory protocols in protocols.io to enhance the reproducibility of your results. Protocols.io assigns your protocol its own identifier (DOI) so that it can be cited independently in the future. For instructions see: https://journals.plos.org/plosone/s/submission-guidelines#loc-laboratory-protocols. Additionally, PLOS ONE offers an option for publishing peer-reviewed Lab Protocol articles, which describe protocols hosted on protocols.io. Read more information on sharing protocols at https://plos.org/protocols?utm_medium=editorial-email&utm_source=authorletters&utm_campaign=protocols.

As the corresponding author, your ORCID iD is verified in the submission system and will appear in the published article. PLOS supports the use of ORCID, and we encourage all coauthors to register for an ORCID iD and use it as well. Please encourage your coauthors to verify their ORCID iD within the submission system before final acceptance, as unverified ORCID iDs will not appear in the published article. *Only*  the individual author can complete the verification step; PLOS staff *cannot*  verify ORCID iDs on behalf of authors.

We look forward to receiving your revised manuscript.

Kind regards,

Paul Obeng, MEd, MSc., M.Phil.

Academic Editor

PLOS One

Journal Requirements:

When submitting your revision, we need you to address these additional requirements.

1. Please ensure that your manuscript meets PLOS ONE's style requirements, including those for file naming. The PLOS ONE style templates can be found at

https://journals.plos.org/plosone/s/file?id=wjVg/PLOSOne_formatting_sample_main_body.pdf and https://journals.plos.org/plosone/s/file?id=ba62/PLOSOne_formatting_sample_title_authors_affiliations.pdf

2. Please include a complete copy of PLOS’ questionnaire on inclusivity in global research in your revised manuscript. Our policy for research in this area aims to improve transparency in the reporting of research performed outside of researchers’ own country or community. The policy applies to researchers who have travelled to a different country to conduct research, research with Indigenous populations or their lands, and research on cultural artefacts. The questionnaire can also be requested at the journal’s discretion for any other submissions, even if these conditions are not met.  Please find more information on the policy and a link to download a blank copy of the questionnaire here: https://journals.plos.org/plosone/s/best-practices-in-research-reporting. Please upload a completed version of your questionnaire as Supporting Information when you resubmit your manuscript.

3. Your ethics statement should only appear in the Methods section of your manuscript. If your ethics statement is written in any section besides the Methods, please delete it from any other section.

4. Please remove your figures from within your manuscript file, leaving only the individual TIFF/EPS image files, uploaded separately. These will be automatically included in the reviewers’ PDF.

5. If the reviewer comments include a recommendation to cite specific previously published works, please review and evaluate these publications to determine whether they are relevant and should be cited. There is no requirement to cite these works unless the editor has indicated otherwise.

6. Please review your reference list to ensure that it is complete and correct. If you have cited papers that have been retracted, please include the rationale for doing so in the manuscript text, or remove these references and replace them with relevant current references. Any changes to the reference list should be mentioned in the rebuttal letter that accompanies your revised manuscript. If you need to cite a retracted article, indicate the article’s retracted status in the References list and also include a citation and full reference for the retraction notice.

[Note: HTML markup is below. Please do not edit.]

Reviewers' comments:

Reviewer's Responses to Questions

**Comments to the Author**

1. Is the manuscript technically sound, and do the data support the conclusions?

Reviewer #1: Yes

Reviewer #2: Yes

**********

2. Has the statistical analysis been performed appropriately and rigorously?

Reviewer #1: Yes

Reviewer #2: No

**********

3. Have the authors made all data underlying the findings in their manuscript fully available?

The PLOS Data policy

Reviewer #1: Yes

Reviewer #2: Yes

**********

4. Is the manuscript presented in an intelligible fashion and written in standard English?

Reviewer #1: No

Reviewer #2: Yes

**********

Reviewer #1: 1. The author presents data repeatedly in different formats. Graphical presentation is unnecessary because it is already presented in tables.

2. Are the findings of this study similar or different from those found in other locations with similar socioeconomic characteristics?

3. Inconsistencies in the use of language style are found. Use an editor to ensure grammatical accuracy.

Reviewer #2: Adolescent sexual and reproductive health remains a significant concern in public health, especially due to teenage pregnancies, unsafe abortions, STIs and HIV&AIDS. This study is important and timely as it explores determinants of risky sexual behaviours, including those that do not frequently feature in published literature i.e. digital media exposure.

**********

what does this mean?). If published, this will include your full peer review and any attached files.

If you choose “no”, your identity will remain anonymous but your review may still be made public.

**Do you want your identity to be public for this peer review?**  For information about this choice, including consent withdrawal, please see our Privacy Policy

Reviewer #1: No

Reviewer #2: No

**********

[NOTE: If reviewer comments were submitted as an attachment file, they will be attached to this email and accessible via the submission site. Please log into your account, locate the manuscript record, and check for the action link "View Attachments". If this link does not appear, there are no attachment files.]

To ensure your figures meet our technical requirements, please review our figure guidelines: https://journals.plos.org/plosone/s/figures

You may also use PLOS’s free figure tool, NAAS, to help you prepare publication quality figures: https://journals.plos.org/plosone/s/figures#loc-tools-for-figure-preparation.

NAAS will assess whether your figures meet our technical requirements by comparing each figure against our figure specifications.

Attachment Submitted filename: Review comments_PONE-D-25-56219_RSB.docx

# Author comment: Author response to Decision Letter 1

**DOI:** 10.1371/journal.pone.0335879.r002

4 May 2026

Response to Reviewer

I sincerely thank the reviewers for the constructive and encouraging comments on my manuscript. I have carefully revised the manuscript in response to each comment. The main revisions include strengthening the policy context, clarifying classroom-based data collection, adding sex-disaggregated descriptive analyses, correcting Table 3 estimates, expanding the Discussion to compare our findings with studies from sub-Saharan Africa and LMICs, and adding a Strengths and Limitations section.

Comment 1

Line 47: Policy commitments on adolescent sexual and reproductive health are acknowledged as existing but these are not highlighted. Please highlight all existing policy commitments, especially in Togo.

Line 48-51: I revised the Introduction to more explicitly mention relevant regional and national policy commitments. Specifically, I now refer to the African Charter on the Rights and Welfare of the Child and Togo’s Code de l’Enfant (Law No. 2007–017), which promotes the protection of children’s rights and defines a child as any person under 18 years. I also retained references to national adolescent pregnancy-related policy efforts and data from Togo.

Comment 2

Lines 147–149: The survey questionnaire was administered to participants in a classroom under supervision to ensure privacy and minimize peer influence. Were all students in a classroom respondents? This needs to be clarified in the manuscript. Second, a classroom does not confer adequate privacy for respondents due to tendency of students to “peep through” or attempts to “gauge” who is ahead in completing the questionnaire. This needs to be acknowledged as a limitation under study limitations.

Line 181-183: I revised the Methods section to clarify that only students selected through the sampling procedure and who provided consent or assent completed the questionnaire.

Line 397-417: I also acknowledged in the limitations that classroom-based data collection may not have ensured complete privacy, despite anonymous completion and sealed-envelope submission.

Comment 3

This study did not look into the self-reported outcomes of risky sexual behaviours, for example unwanted pregnancies, STIs, type of sex, and contraceptive use. I suggest that these be highlighted and included under study limitations.

Line 397-417: I added this point to the Strengths and Limitations section. The revised limitation now explicitly states that the study did not assess unintended pregnancy, pregnancy termination, current or previous STIs, type of sexual intercourse, or broader contraceptive use beyond condom-use consistency.

Comment 4

Did both sexes exhibit similar or different sexual behaviours? The analysis would be more informative if it was disaggregated by sex.

I revised Table 2 to present sex-disaggregated descriptive results for all key sexual behaviour variables. The revised Results section now highlights gendered patterns, including differences in past-year sexual activity, coerced first sex, partner age difference, transactional sex, and casual sexual encounters. I also expanded the Discussion to interpret these findings in relation to gendered power dynamics and sexual negotiation.

Fully sex-stratified multivariable models were explored but were limited by sparse cells and wide confidence intervals. I therefore retained the pooled multivariable model as the main analysis and noted this limitation.

Comment 5

Line 273/Table 3: The confidence intervals stated for agemates in the household and religion do not match the stated AORs. Please recheck the analysis.

I rechecked the regression outputs and corrected Table 3. The adjusted odds ratios and confidence intervals now align correctly. For example, the AOR for having one to two agemates in the household is now reported as 0.23 with a 95% CI of 0.05–0.98. For religion, the revised table reports Islam as AOR = 0.44, 95% CI: 0.08–2.25, and other religions as AOR = 0.07, 95% CI: 0.01–0.37.

Comment 6

Lines 278–382/Discussion section: How do findings of this study compare and contrast with other similar studies, especially those conducted within the same region or LMICs? Are the identified determinants of RSB similar or different and what are the most probable explanations?

Line 292-387: I substantially revised the Discussion to compare and contrast the findings with evidence from sub-Saharan Africa and LMICs. I discuss how early sexual initiation, multiple partnerships, coerced first sex, and digital exposure align with or differ from previous studies. I also provide possible explanations for findings that differed from prior literature, particularly the association between later sexual debut and RSB. In addition, I revised the language from “determinants” to “associated factors” to avoid causal overstatement given the cross-sectional design.

Comment 7

Study limitations section is missing. Please include.

Line 397-417: I have added a dedicated “Strengths and limitations of the study” section. This section summarizes the study’s strengths and acknowledges limitations related to cross-sectional design, self-reporting, classroom-based data collection, the 12-month definition of sexual activity, unmeasured SRH outcomes, the composite RSB measure, and limited power for fully sex-stratified multivariable analyses.

Attachment Submitted filename: Response to Reviewers.docx

# Peer review: Decision Letter 1

**DOI:** 10.1371/journal.pone.0335879.r003

**Authors:** Adewale Ashimi

10 Jul 2026

Dear Dr. Kpegba-Fiaboe,

Thank you for submitting your manuscript to PLOS ONE. After careful consideration, we feel that it has merit but does not fully meet PLOS ONE’s publication criteria as it currently stands. Therefore, we invite you to submit a revised version of the manuscript that addresses the points raised during the review process.

Please submit your revised manuscript by Aug 24 2026 11:59PM. If you will need more time than this to complete your revisions, please reply to this message or contact the journal office at plosone@plos.org. When you're ready to submit your revision, log on to https://www.editorialmanager.com/pone/ and select the 'Submissions Needing Revision' folder to locate your manuscript file.

- A letter that responds to each point raised by the academic editor and reviewer(s). You should upload this letter as a separate file labeled 'Response to Reviewers'.
- A marked-up copy of your manuscript that highlights changes made to the original version. You should upload this as a separate file labeled 'Revised Manuscript with Track Changes'.
- An unmarked version of your revised paper without tracked changes. You should upload this as a separate file labeled 'Manuscript'.

If you would like to make changes to your financial disclosure, please include your updated statement in your cover letter. Guidelines for resubmitting your figure files are available below the reviewer comments at the end of this letter.

If applicable, we recommend that you deposit your laboratory protocols in protocols.io to enhance the reproducibility of your results. Protocols.io assigns your protocol its own identifier (DOI) so that it can be cited independently in the future. For instructions see: https://journals.plos.org/plosone/s/submission-guidelines#loc-laboratory-protocols. Additionally, PLOS ONE offers an option for publishing peer-reviewed Lab Protocol articles, which describe protocols hosted on protocols.io. Read more information on sharing protocols at https://plos.org/protocols?utm_medium=editorial-email&utm_source=authorletters&utm_campaign=protocols.

As the corresponding author, your ORCID iD is verified in the submission system and will appear in the published article. PLOS supports the use of ORCID, and we encourage all coauthors to register for an ORCID iD and use it as well. Please encourage your coauthors to verify their ORCID iD within the submission system before final acceptance, as unverified ORCID iDs will not appear in the published article. *Only*  the individual author can complete the verification step; PLOS staff *cannot*  verify ORCID iDs on behalf of authors.

We look forward to receiving your revised manuscript.

Kind regards,

Adewale Olufemi Ashimi, MBChB, MPH, PhD, FWACS

Academic Editor

PLOS One

Journal Requirements:

If the reviewer comments include a recommendation to cite specific previously published works, please review and evaluate these publications to determine whether they are relevant and should be cited. There is no requirement to cite these works unless the editor has indicated otherwise.

Please review your reference list to ensure that it is complete and correct. If you have cited papers that have been retracted, please include the rationale for doing so in the manuscript text, or remove these references and replace them with relevant current references. Any changes to the reference list should be mentioned in the rebuttal letter that accompanies your revised manuscript. If you need to cite a retracted article, indicate the article’s retracted status in the References list and also include a citation and full reference for the retraction notice.

[Note: HTML markup is below. Please do not edit.]

Reviewers' comments:

Reviewer's Responses to Questions

**Comments to the Author**

Reviewer #2: All comments have been addressed

Reviewer #3: (No Response)

**********

2. Is the manuscript technically sound, and do the data support the conclusions?

Reviewer #2: Yes

Reviewer #3: Yes

**********

3. Has the statistical analysis been performed appropriately and rigorously?

Reviewer #2: Yes

Reviewer #3: Yes

**********

4. Have the authors made all data underlying the findings in their manuscript fully available?

The PLOS Data policy

Reviewer #2: No

Reviewer #3: Yes

**********

5. Is the manuscript presented in an intelligible fashion and written in standard English?

Reviewer #2: Yes

Reviewer #3: Yes

**********

Reviewer #2: The second full document review confirms that all comments were satisfactorily addressed. Particularly, it is noteworthy that the reviewer recommendation on gendered-disaggregated descriptive analysis unearthed important differences.

Reviewer #3: This is a very good research topic and is of great importance in this era of increasing digital influence especially amongst young people and adolescents.

The manuscript has also been very well written and I surely enjoyed reading it. Below are just a few minor things in the methods section that could be done to improve your work.

I) Study design: All cross sectional studies are quantitative so there is no need to say cross sectional quantitative study

ii) Eligibility criteria: your exclusion criteria does not seem to make much sense, because the people you are excluding were already not supposed to be included e.g. the age group and not being in the selected schools. These are already not part of your study population. So you should only exclude those who would have otherwise been part of your study, but because of certain factors, are unable to participate e.g. sickness, language barrier etc.

•

**********

what does this mean?). If published, this will include your full peer review and any attached files.

If you choose “no”, your identity will remain anonymous but your review may still be made public.

**Do you want your identity to be public for this peer review?**  For information about this choice, including consent withdrawal, please see our Privacy Policy

Reviewer #2: **Yes:**  Charles Muruka

Reviewer #3: No

**********

[NOTE: If reviewer comments were submitted as an attachment file, they will be attached to this email and accessible via the submission site. Please log into your account, locate the manuscript record, and check for the action link "View Attachments". If this link does not appear, there are no attachment files.]

To ensure your figures meet our technical requirements, please review our figure guidelines: https://journals.plos.org/plosone/s/figures

You may also use PLOS’s free figure tool, NAAS, to help you prepare publication quality figures: https://journals.plos.org/plosone/s/figures#loc-tools-for-figure-preparation.

NAAS will assess whether your figures meet our technical requirements by comparing each figure against our figure specifications.

# Author comment: Author response to Decision Letter 2

**DOI:** 10.1371/journal.pone.0335879.r004

10 Jul 2026

I sincerely thank the reviewers for their careful evaluation of the manuscript and their constructive feedback. The responses below describe the revisions made to address each comment.

Reviewer #2

Comment:

The second full document review confirms that all comments were satisfactorily addressed. Particularly, it is noteworthy that the reviewer recommendation on gender-disaggregated descriptive analysis unearthed important differences.

Response:

I sincerely thank the reviewer for confirming that the previous comments were satisfactorily addressed. I also appreciate the recommendation to present sex-disaggregated descriptive analyses, which helped identify important differences between male and female students and strengthened the interpretation of the findings.

Reviewer #3

General comment:

This is a very good research topic and is of great importance in this era of increasing digital influence, especially among young people and adolescents. The manuscript is also very well written.

Response:

I thank the reviewer for the positive assessment of the study topic and the quality of the manuscript. I also appreciate the helpful suggestions provided to improve the Methods section.

Comment I – Study design:

All cross-sectional studies are quantitative, so there is no need to describe the study as a “cross-sectional quantitative study.”

Response:

I agree with the reviewer. The wording has been revised from “cross-sectional quantitative design” to “cross-sectional design” in the Methods section under the subsection “Study design and setting.”

Comment II – Eligibility criteria:

The exclusion criteria are redundant because students outside the specified age range or those not enrolled in the selected schools were already outside the study population. Exclusion criteria should apply only to individuals who otherwise meet the inclusion criteria but are unable to participate because of factors such as illness or a language barrier.

Response:

I agree with the reviewer and have revised the “Eligibility criteria” subsection accordingly. The redundant exclusion criteria relating to age and enrolment in the selected schools have been removed. The revised text now clarifies that otherwise eligible students were excluded if illness prevented them from completing the questionnaire, if they declined to participate, or if they withdrew their consent or assent.

Attachment Submitted filename: Response_to_Reviewers_auresp_2.docx

# Peer review: Decision Letter 2

**DOI:** 10.1371/journal.pone.0335879.r005

**Authors:** Adewale Ashimi

21 Jul 2026

Risky sexual behaviours and associated factors among high school students in Lomé, Togo: a cross-sectional study

PONE-D-25-56219R2

Dear Dr. Kpegba-Fiaboe,

We’re pleased to inform you that your manuscript has been judged scientifically suitable for publication and will be formally accepted for publication once it meets all outstanding technical requirements.

Within one week, you’ll receive an e-mail detailing the required amendments. When these have been addressed, you’ll receive a formal acceptance letter and your manuscript will be scheduled for publication.

An invoice will be generated when your article is formally accepted. Please note, if your institution has a publishing partnership with PLOS and your article meets the relevant criteria, all or part of your publication costs will be covered. Please make sure your user information is up-to-date by logging into Editorial Manager at Editorial Manager® and clicking the ‘Update My Information' link at the top of the page. For questions related to billing, please contact billing support.

If your institution or institutions have a press office, please notify them about your upcoming paper to help maximize its impact. If they’ll be preparing press materials, please inform our press team as soon as possible -- no later than 48 hours after receiving the formal acceptance. Your manuscript will remain under strict press embargo until 2 pm Eastern Time on the date of publication. For more information, please contact onepress@plos.org.

Kind regards,

Adewale Olufemi Ashimi, MBChB, MPH, PhD, FWACS

Academic Editor

PLOS One

Additional Editor Comments (optional):

Reviewers' comments:

Reviewer's Responses to Questions

**Comments to the Author**

Reviewer #3: All comments have been addressed

**********

2. Is the manuscript technically sound, and do the data support the conclusions?

Reviewer #3: Yes

**********

3. Has the statistical analysis been performed appropriately and rigorously?

Reviewer #3: Yes

**********

4. Have the authors made all data underlying the findings in their manuscript fully available?

The PLOS Data policy

Reviewer #3: Yes

**********

5. Is the manuscript presented in an intelligible fashion and written in standard English?

Reviewer #3: Yes

**********

Reviewer #3: (No Response)

**********

what does this mean?). If published, this will include your full peer review and any attached files.

If you choose “no”, your identity will remain anonymous but your review may still be made public.

**Do you want your identity to be public for this peer review?**  For information about this choice, including consent withdrawal, please see our Privacy Policy

Reviewer #3: No

**********

# Editor report: Acceptance letter

**DOI:** 10.1371/journal.pone.0335879.r006

**Authors:** Adewale Ashimi

PONE-D-25-56219R2

PLOS One

Dear Dr. Kpegba-Fiaboe,

I'm pleased to inform you that your manuscript has been deemed suitable for publication in PLOS One. Congratulations! Your manuscript is now being handed over to our production team.

At this stage, our production department will prepare your paper for publication. This includes ensuring the following:

* All references, tables, and figures are properly cited

* All relevant supporting information is included in the manuscript submission,

* There are no issues that prevent the paper from being properly typeset

You will receive further instructions from the production team, including instructions on how to review your proof when it is ready. Please keep in mind that we are working through a large volume of accepted articles, so please give us a few days to review your paper and let you know the next and final steps.

Lastly, if your institution or institutions have a press office, please let them know about your upcoming paper now to help maximize its impact. If they'll be preparing press materials, please inform our press team within the next 48 hours. Your manuscript will remain under strict press embargo until 2 pm Eastern Time on the date of publication. For more information, please contact onepress@plos.org.

You will receive an invoice from PLOS for your publication fee after your manuscript has reached the completed accept phase. If you receive an email requesting payment before acceptance or for any other service, this may be a phishing scheme. Learn how to identify phishing emails and protect your accounts at https://explore.plos.org/phishing.

If we can help with anything else, please email us at customercare@plos.org.

Thank you for submitting your work to PLOS ONE and supporting open access.

Kind regards,

PLOS ONE Editorial Office Staff

on behalf of

Dr. Adewale Olufemi Ashimi

Academic Editor

PLOS One
